# Long lifetime of bialkali photocathodes operating in high gradient superconducting radio frequency gun

**DOI:** 10.1038/s41598-021-83997-1

**Published:** 2021-02-24

**Authors:** E. Wang, V. N. Litvinenko, I. Pinayev, M. Gaowei, J. Skaritka, S. Belomestnykh, I. Ben-Zvi, J. C. Brutus, Y. Jing, J. Biswas, J. Ma, G. Narayan, I. Petrushina, O. Rahman, T. Xin, T. Rao, F. Severino, K. Shih, K. Smith, G. Wang, Y. Wu

**Affiliations:** 1 Collider-Accelerator Department, Brookhaven National Laboratory, Upton, NY 11973 USA; 2 Department of Physics and Astronomy, Stony Brook University, Stony Brook, NY 11794 USA; 3 Fermi National Accelerator Laboratory, Batavia, IL 60510 USA; 4 Instrumentation Division, Brookhaven National Laboratory, Upton, NY 11973 USA; 5 Institute of High Energy Physics, Chinese Academy of Sciences, Beijing, 100049 China

**Keywords:** Materials for devices, Techniques and instrumentation, Superconducting devices

## Abstract

High brightness, high charge electron beams are critical for a number of advanced accelerator applications. The initial emittance of the electron beam, which is determined by the mean transverse energy (MTE) and laser spot size, is one of the most important parameters determining the beam quality. The bialkali photocathodes illuminated by a visible laser have the advantages of high quantum efficiency (QE) and low MTE. Furthermore, Superconducting Radio Frequency (SRF) guns can operate in the continuous wave (CW) mode at high accelerating gradients, e.g. with significant reduction of the laser spot size at the photocathode. Combining the bialkali photocathode with the SRF gun enables generation of high charge, high brightness, and possibly high average current electron beams. However, integrating the high QE semiconductor photocathode into the SRF guns has been challenging. In this article, we report on the development of bialkali photocathodes for successful operation in the SRF gun with months-long lifetime while delivering CW beams with nano-coulomb charge per bunch. This achievement opens a new era for high charge, high brightness CW electron beams.

## Introduction

High brightness, high current electron beam is the key towards many future critical accelerator facilities and applications, such as the hadron cooling^1,2^, hard X-ray CW free electron lasers^3^, ERL-based extreme ultraviolet lithography machines^4,5^, high intensity, high flux gamma ray sources^6,7^, ultrafast electron diffraction microscope. When the 6D phase space volume is preserved in the beam transport, the quality of electron beams is fundamentally determined by its initial brightness at the source.

The initial average 4-D brightness of electron beam is determined by the photocathode properties and electric field gradient on photocathode at the moment of emission (${E}_{cath}$) and can be expressed as^8^

1 $$B\propto f\frac{{E}_{cath}}{MTE},$$

where the MTE is mean transverse energy of the electrons, *f* is the repetition rate of the beam. Therefore, a photocathode with low MTE operating in a high gradient gun offers a clear path towards high brightness electron beams.

A quarter-wave (QW) SRF gun, with a modest frequency of ~ 100 MHz, has several advantages including operating with high gradient at cathode at the optimum emission phase approaching the crest of the accelerating field. It can also operate at CW mode generating stable and high average current electron beam^9–13^. Such SRF gun operates with much higher gradient and generates much higher beam energy when compared with the DC guns^14,15^. SRF gun can generate higher accelerating gap voltage, compared to CW mode very high frequency normal conducting (VHF-NC) RF guns, whose gap voltage is limited by cooling capability and heat induced outgassing^14^. SRF guns can easily be used to operate cathode in cryogenic temperatures that will generate extremely low MTE beam^16^.

The bialkali antimonide photocathodes, which have 10% electron yield at green light, can produce electron beams with smaller emittance than other semiconductor photocathodes (such as Cs_2_Te) and metal photocathodes driven by UV lasers. The MTE can be estimated by the following equation^17^

2 $$MTE\propto \frac{h\nu -\left(\varphi -e\sqrt{\frac{eE}{4\pi {\epsilon }_{0}}}\right)}{3} ,$$

where $\varphi$ is the work function, $e\sqrt{eE/4\pi {\epsilon }_{0}}$ is the reduction in the work function due to Schottky effect in the presence of the applied field gradient *E*, and $h\nu$ is the photon energy. For the same accelerating gradient, at typical photon energies used with semiconductor cathodes as electron sources, bialkali antimonide photocathode shows smaller thermal emittance than Cs_2_Te photocathode^14,18^. Semiconductor and metal photocathodes have been tested in a number of electron guns. Until our SRF gun became operational, the bialkali photocathodes were successfully operated only in the high voltage (HV) DC guns^19,20^, generating tens of milliamperes of average current with lifetime of the order of months^21^. However, the performance of alkali cathodes in RF guns were less successful. Tests of bialkali photocathode in the normal conducting APEX NCRF gun showed a short lifetime due to the heating and residual gas pressure increase^18^. The tests of Cs_2_Te photocathode in their 2nd 1.3 GHz SRF gun at Helmholtz-Zentrum Dresden-Rossendorf were also unsuccessful, resulting in the use of magnesium cathode^22,23^ ultimately. Although Cs_2_Te is much more robust than the bialkali photocathodes, its short lifetime was explained by excessive heating by the 1.3 GHz RF field ^23^.

Degradation of bialkali cathodes by residual gases in normal conducting RF guns has so far hampered their use in CW mode of operation. SRF guns have the important advantage of ultra-high vacuum environment because of the inherent cryo-pumping. However, operating high QE bialkali photocathode in SRF gun has the following challenges:

1. Possibility that multipacting can induce gas desorption from the cavity walls and poison the photocathode;
2. Need for maintaining the room temperature of the photocathode inside cryogenic SRF gun environment;
3. Possibility of evaporated photocathode materials poisoning the SRF cavity either by reducing its quality factor or generating dark current emission centers;
4. Possibility of field emission caused by the high field gradient and low work-function of the cathode.

In this article, we report on the long lifetime operation of K-Cs-Sb photocathode in the 113 MHz CW SRF gun. We also report on our growth recipe to produce cathodes with 10% QE within 2 h. We present details on all important cathode-related issues including modification of cathode area, design of cathode insertion stalk, vacuum estimation and selection of cathode gradient.

As reported previously^11^, our 113 MHz SRF gun with bialkali photocathode generated record low normalized beam emittances: 0.3 mm-mrad with100 pC charge per bunch. Based on these performances, the combination of bialkali photocathode illuminated by a visible laser and the QW SRF electron gun can be considered as the best candidate to generate the highest brightness of high current CW electron beams.

## Methods

**Figure 1:**
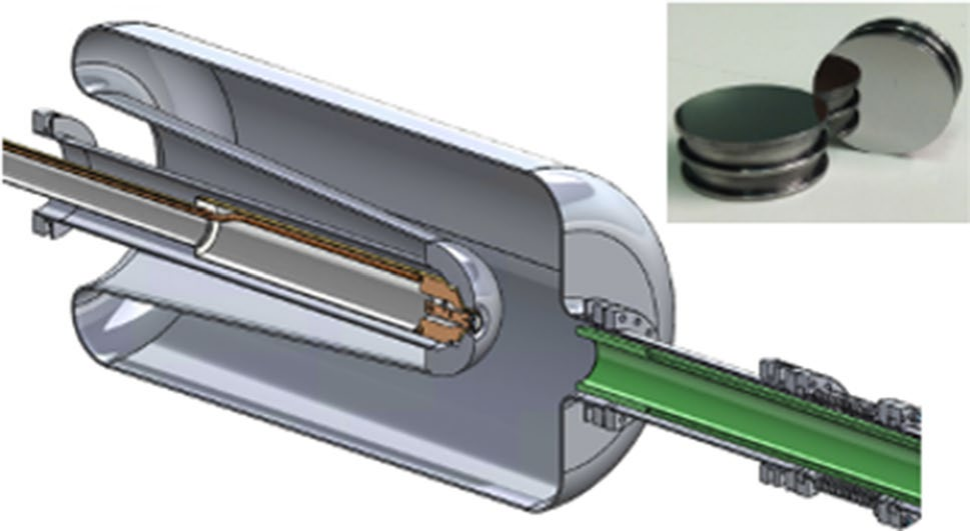
SRF gun 3D model. The 3D model of the 113 MHz SRF gun with cathode stalk (brown color, left) and the axial fundamental power coupler (FPC, green color, right). Inserted photograph shows two mirror-polished molybdenum substrates (pucks) used for photocathode deposition.

The 1.25 meV 113 MHz QW SRF gun, located at Interaction Region 2 of the Relativistic Heavy Ion Collider (RHIC), has been designed and built as the CW electron beam source for the Coherent electron Cooling Proof of Principle (CeC-PoP) experiment at Brookhaven National Laboratory (BNL)^24^. The gun cavity, shown in Fig. 1, was designed by BNL and fabricated by Niowave Inc. The photocathode stalk insertion system and cathode related components were designed and fabricated by BNL and Department of Physics and Astronomy at Stony Brook University (SBU). Detailed description of the gun RF design and its performance can be found in reference^25^ .

### Bialkali photocathodes preparation

The K–Cs–Sb photocathode is deposited on an arc-cast molybdenum (Mo) substrate puck polished to mirror quality. We chose Mo due to its ability to withstand high stress, its low RF heat losses and its high thermal conductivity. To generate stable high QE K–Cs–Sb photocathode, a rapid sequential cathode growth procedure has been developed.

The cathodes deposition is carried out in an ultra-high vacuum (UHV) system described in reference^26^. The Mo substrates are mechanically polished down to rms roughness of 2 nm and thoroughly cleaned from the polishing residuals and handled to be particulate free before the cathode deposition. Substrates are then heated in the preheating section of the vacuum chamber to 350 °C for 12 h, then cooled down to 80 °C for cathode deposition. First, a 10 nm thick Sb film is deposited at a rate of 0.5–2 Å/s, which is measured using quartz crystal monitor. The substrate is then heated up to 135 °C in less than 5 min for K deposition with the rate is 0.5 Å/s. The ratio of Sb and K is optimized to maximize the final QE. We found that the optimal thickness of K should be 2.2 times the thickness of Sb. During this process, we measure the photocurrent from the K–Sb bilayer and terminate K deposition at the first appearance of detectable photocurrent.

At the completion of K deposition, the substrate is let to gradually cool down and Cs deposition begins during this cooling phase. The Cs evaporation is stopped once substrate temperature drops below 75 °C, which is the known temperature for maintaining the stable stoichiometry of K–Cs–Sb. After a couple of hours of photocathode stabilization, the typical QE is ~ 10%.

The entire duration of our cathode growth process is about 2.5 h and is at least a factor of 2–5 times faster than that reported by others^27–29^. Our cathodes, produced using this recipe, have demonstrated several weeks lifetime while delivering tens of milliamperes of average current in the DC gun^21^.

**Figure 2:**
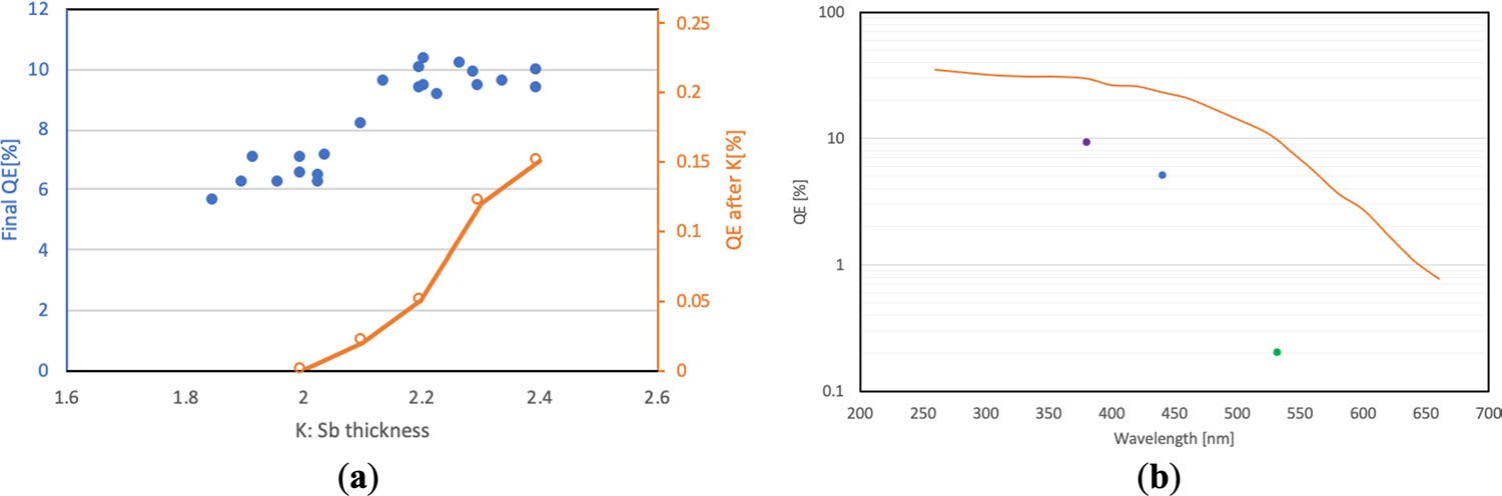
Photocathodes growth optimization. (**a**) Evolution of the QE during the K deposition (orange circles and curve) and the final QE (blue circles) as the function of the K to Sb layer thickness ratio; (**b**) the spectral response of freshly prepared cathode (orange curve) and that of the used cathode retracted from the SRF gun (dots).

The key for producing a stable, high-QE photocathode is controlling the thickness of the K layer. Figure 2a shows the final QE and QE during the K deposition as the function of ratio of thicknesses of K and Sb layers. The final QE nearly plateaus when the ratio reaches value of 2.2, and further deposition of K will result in no QE improvement but only in a longer deposition time.

When the ratio of the K and Sb layer thicknesses approaches to 3:1, formation of K_3_Sb will prevent diffusion of Cs into the K_3_Sb crystal causing much slower generation of K–Cs–Sb crystalline structures. It is critical to initiate Cs evaporation and start producing the K–Cs–Sb crystal structure prior to the K–Sb crystal generation.

The measured spectral response of prepared cathode is shown in Fig. 2b.

### Quarter-wave SRF gun with bialkali photocathodes

#### Cathodes transferring

**Figure 3:**
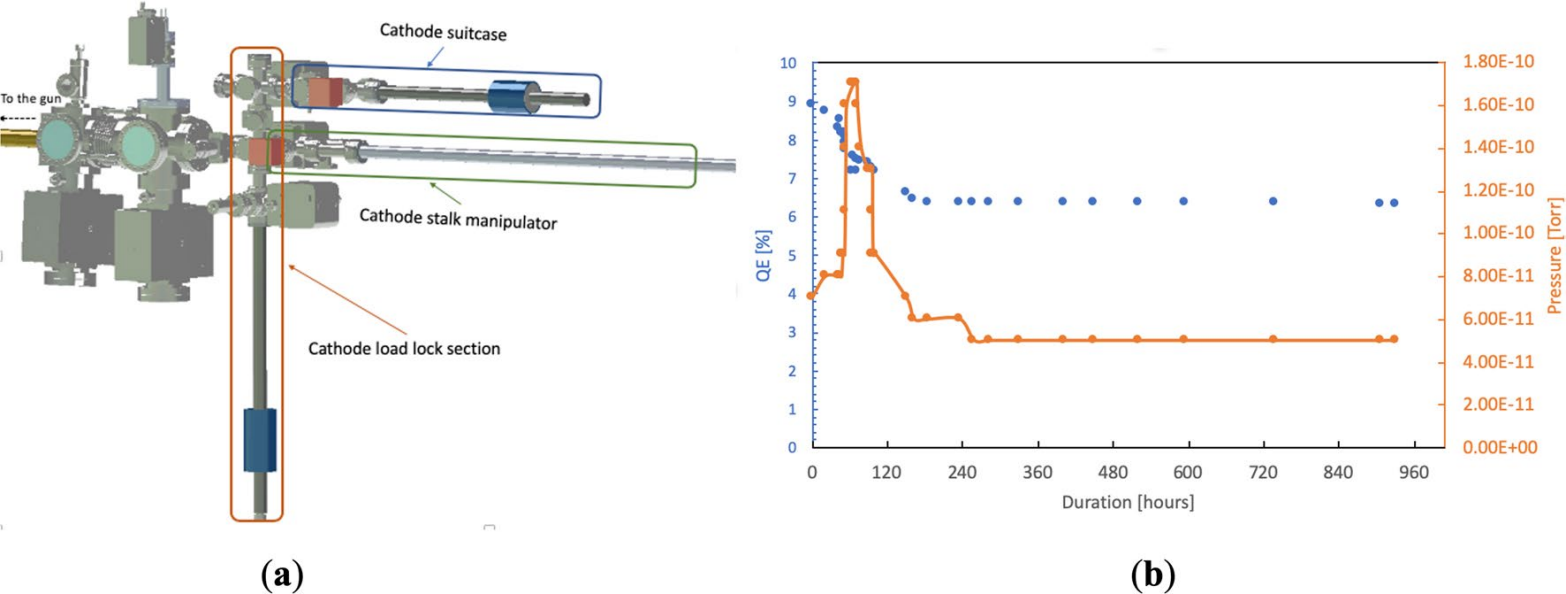
Photocathodes transferring. (**a**) The cathode portable suitcase and its assembly to the SRF gun cathode transfer system; (**b**) the cathode QE evolution during the cathode transfer from the growth chamber to the SRF gun.

The cathode deposition system is housed in a research building about a mile away from the SRF gun which is located in the RHIC tunnel. We designed and built two identical portable UHV suitcases to transfer the photocathodes from the production site to the SRF gun. Each suitcase can accommodate up to 3 cathodes. The suitcase includes a manipulator, a cold cathode gauge, a SAES NEXTorr non evaporable getter (NEG) pump and an anode for continuous QE monitoring. The baseline vacuum pressure of 8 × 10^–11^ torr, with less than 10^–12^ torr of water partial pressure, can be achieved in the suitcase after 2 days of baking. The load-lock, attached to the SRF gun cathode transfer system (see Fig. 3**a**), is baked for 52 h at 200 °C after the suitcase installation. The photocathode’s QE is monitored during the transfer and the load-lock bakeout. Figure 3b illustrates the photocathode QE evolution during its transport and storage in the cathode suitcase. During the bake-out and the temperature ramp up, the valve outgassing causes an increase in the base pressure in the suitcase to 1.5 × 10^–10^ torr over a 5-h period, resulting in a QE reduction from 8.5 to 7.5%. During the next 2 days of the bake, the QE gradually stabilized at 6.5%. Without this bake, the QE lifetime of the cathodes in the suitcase is about 2 months. After the bake is completed, the QE does not degrade any further. The QE measurements over a month indicate practically infinite QE lifetime.

The cathode is inserted into the SRF gun through a 2 m long manipulator and locked at the thermally insulated cathode stalk. The cathode temperature is maintained by a 20 °C circulating water through a water channel. The cathode position can be controlled by the manipulator maintaining cathode at room temperature is important to preserve its QE. It is known that the cathode QE will dramatically decrease at the 4 K temperature of the SRF gun environment^26^.

The hollow stainless-steel gold-coated (to reduce IR emission) cathode stalk also serves as a half-wave RF choke while simultaneously thermally insulating the SRF cavity and the cathode transfer and support system. Gold plated spring fingers provide electrical contacts between the cathode holder and the cathode stalk, preventing the RF field from penetrating into the cathode stalk, and help to maintain the cathode at room temperature. The cathode transfer is done at low velocity to maintain the chamber pressure below 2 × 10^–9^ torr. When the pressure exceeded 2 × 10^–9^ torr, we observed a QE degradation. In practice, the cathode exchange takes approximately 30 min, during which the cathode is occasionally exposed to short vacuum spikes of high 10^–9^ torr scale. The resulting slight QE degradation is reversed when the gun starts generating electron beam. This process usually takes few days and brings the QE up back to about 5%.

#### Multipacting

We investigated the effect of the size of the photocathode on the SRF gun operation. When the entire 20 mm diameter of the substrate surface was coated by the photocathode material, we observed strong multipacting in the SRF gun and subsequent outgassing causing the QE to decay within minutes. We attribute this effect to the absorption of ambient photons at the cathode, which generate electrons that initiate the multipacting. Our simulations indicate that free electrons emitted from the cathode edge can trigger the multipacting process when the secondary electron emission yield (SEY) coefficient exceeds 20, a value typical for multialkali photocathodes^30^.

**Figure 4:**
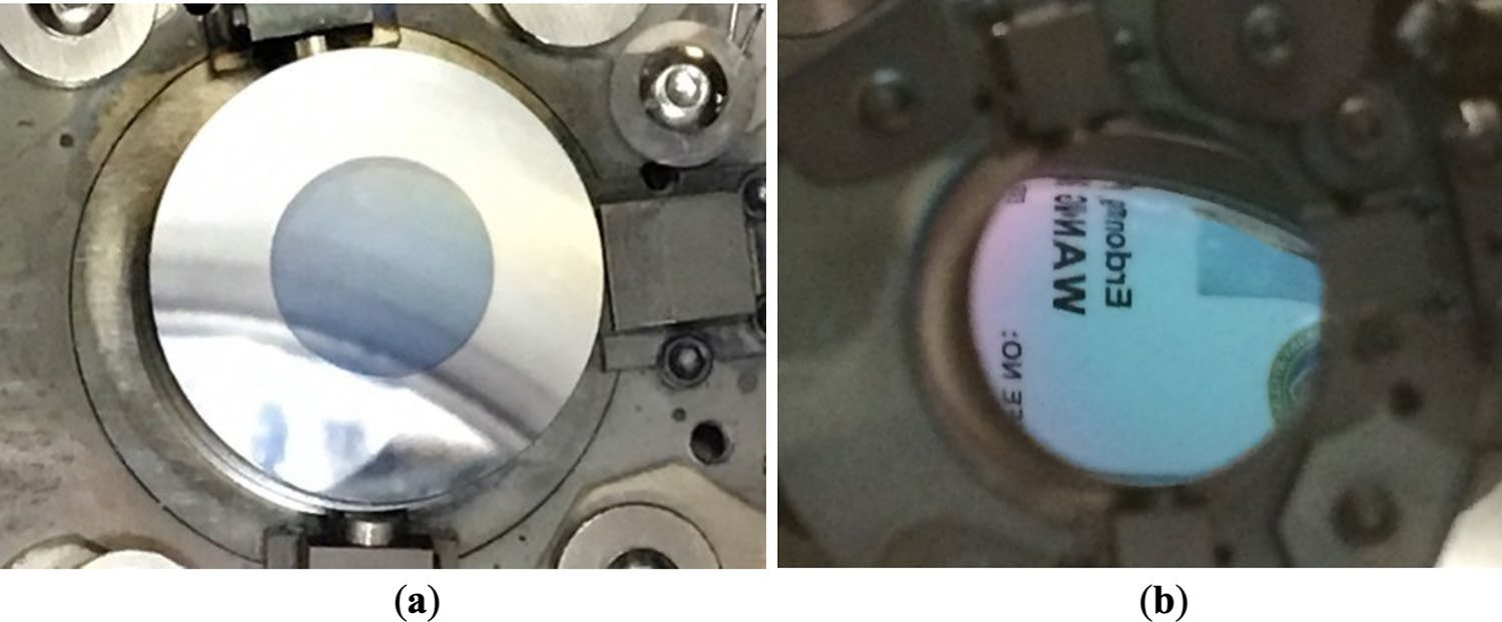
Photocathodes photographs w/o multipacting. Photographs of the photocathode deposited on the puck with use (**a**) and without use (**b**) of a cap shielding the puck edge.

The color of the cathode, when exposed to significant multipacting, changes from light blue to dark grey. This indicates that the alkali material is either oxidized or missing. We eliminated the photocathode initiated SRF gun multipacting by shielding the edges and sides of puck from the deposition of the high SEY cathode material. We are using a shielding cap to coat only the center 9 mm diameter of the cathode substrate with. Figure 4 shows the cathode with (4a) and without (4b) edge shielding.

Initial commissioning of the SRF gun with K–Cs–Sb photocathode was a very rocky trial-and-error process. We observed a number of QE degrading events during the gun commissioning in 2016, which mostly occurred because of the low voltage multipacting during the turn-on processes of the gun. After detailed studies and understanding of the problem, we developed a low level RF control script to automatically detect and to avoid multipacting zones^31^. With mitigated multipacting, the cathode operational lifetime increased typically to a month, and sometimes even longer. The longest serving photocathode provided electron beam with the bunch charge up to 2 nC for more than 6 months.

We noticed that some multipacting events, lasting for a significant amount of time, might cause degradation of the gun performance. Specifically, we observed that frequently the gun cannot start up immediately after a multipacting event has occurred. However, the gun can be restarted and can reach 1.25 MV voltage after a rest period lasting from typically 30 min to occasionally few hours. We attribute this to a multipacting centers caused by deposition high SEY material on the cavity walls.

It is worth mentioning, that with the accelerating gradient in our SRF gun, we typically observe extremally low, e.g. sub-nanoampere, dark current. Nevertheless, in few occasions we observed generation of micro-ampere scale dark currents from the surface on the cathode due either to the cathode puck contamination or sharp edges of the cathode puck. As a result, we developed a thorough procedure for cathode puck cleaning and edge preparation to avoid re-occurrence of such events.

## Results and discussion

The SRF gun is kept at 1.25 MV operational voltage at all times, except for occasional trip and bi-weekly maintenance accesses when all RHIC systems are turned off. Most of the time the system is operated with 1 to 2 nC charge per bunch generated at a 78 kHz repetition rate. Tuning of the CeC system is usually performed with a limited number of bunches, but when needed for the experiment the full power beam with average current of about 150 μA is used.

**Figure 5:**
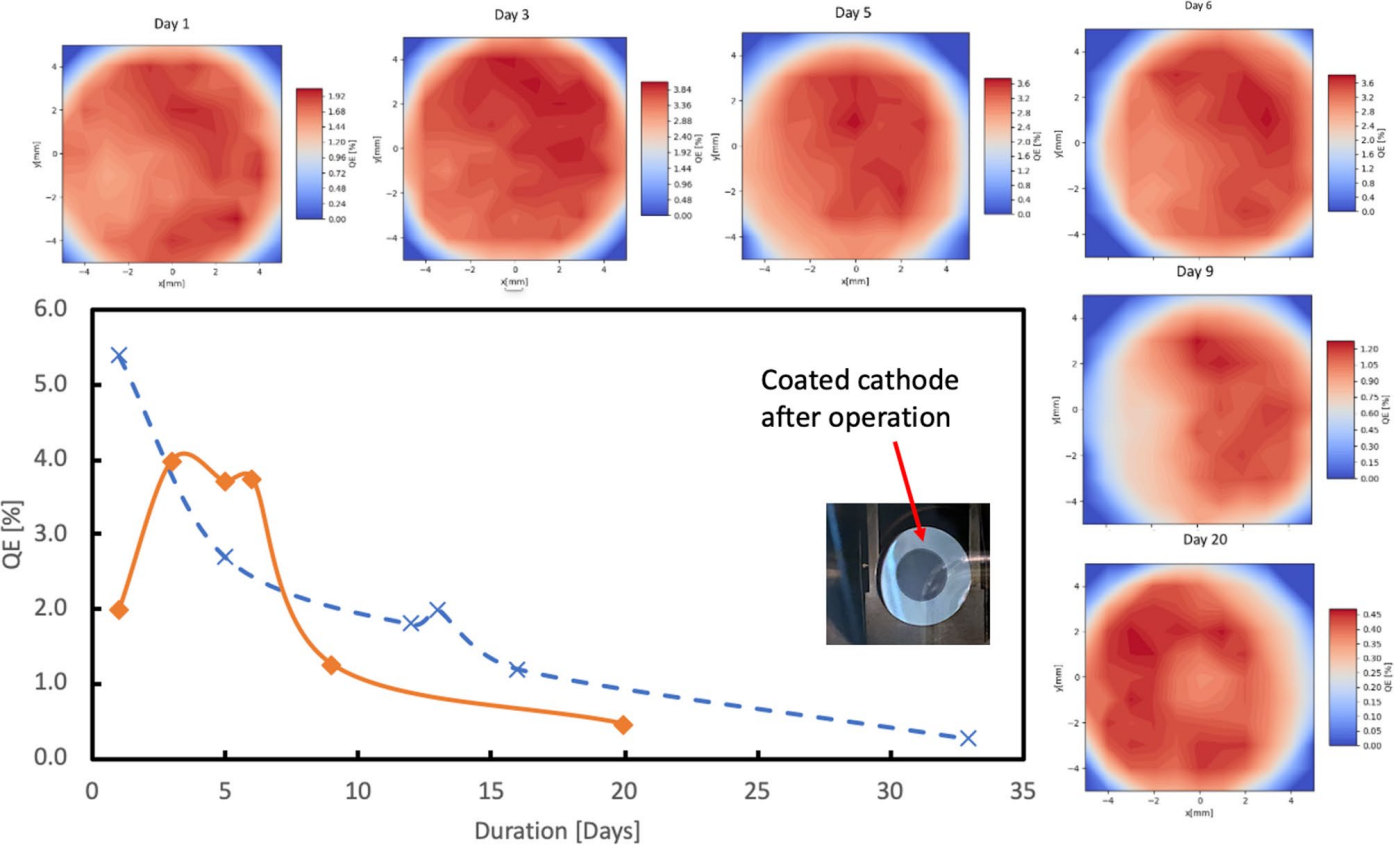
Photocathodes lifetime in operation. The cathode QE evolution in the gun operating in CW RF mode with pulsed beam. Solid orange curve is for the cathode exposed to MP and dashed blue curve is for another cathode, not exposed to MP in the SRF gun. Each QE map response to one data point in the orange curve.

Figure 5 shows monthly QE-map evolution of two cathodes inside the gun. The QEs of both cathodes before delivered into the gun were above 5%. The solid curve in Fig. 5 shows a sharp QE drop due to a MP event. The QE then recovers over the next few days during CW RF gun operation, which we attribute to the elimination of the top contaminated layer by X-rays generated in the gun, and the subsequent exposure of fresh cathode material. Without multipacting, as shown by the dashed curve, the QE was similar to that in the transfer chamber. Then the QE gradually dropped down during 1-month operation. During the operation period of 2017–2019, the average useful cathode lifetime in the SRF gun—the QE dropping to 0.3%—was about a month.

The QE maps were taken periodically as indicated by labels on the top of each maps (see Fig. 5). The typical laser spot size is from 3 to 6 mm in diameter. We did not observe any QE recovery at the location of laser illumination, implying that QE recovery is not caused by cathode heating due to the laser power. Therefore, we concluded that such relatively uniform QE recovery is most likely caused by soft-X-ray radiation generated by the SRF gun. After 20 days of operation, the QE at the center of the cathode was about half of that at the edge. This effect could be caused either by the ion back bombardment or Cs depletion. The photograph of the used cathode is shown in the insert of Fig. 5. We did not observe either the color change or the discharge spots that are usually seen on the cathodes damaged in a DC guns. The color change of the entire cathode to gray indicates that either the thickness of cathode or the cathode stoichiometry has changed. We also measured the QE of the used cathode by illuminating it with 380, 420 and 520 nm laser as shown on Fig. 2b. Although the QE at 530 nm has already dropped to 0.2%, QE values at shorter wavelengths were still at 5–10%. This suggests that using short wavelength laser can significantly increase the cathode lifetime with a tradeoff of higher thermal emittance.

**Figure 6:**
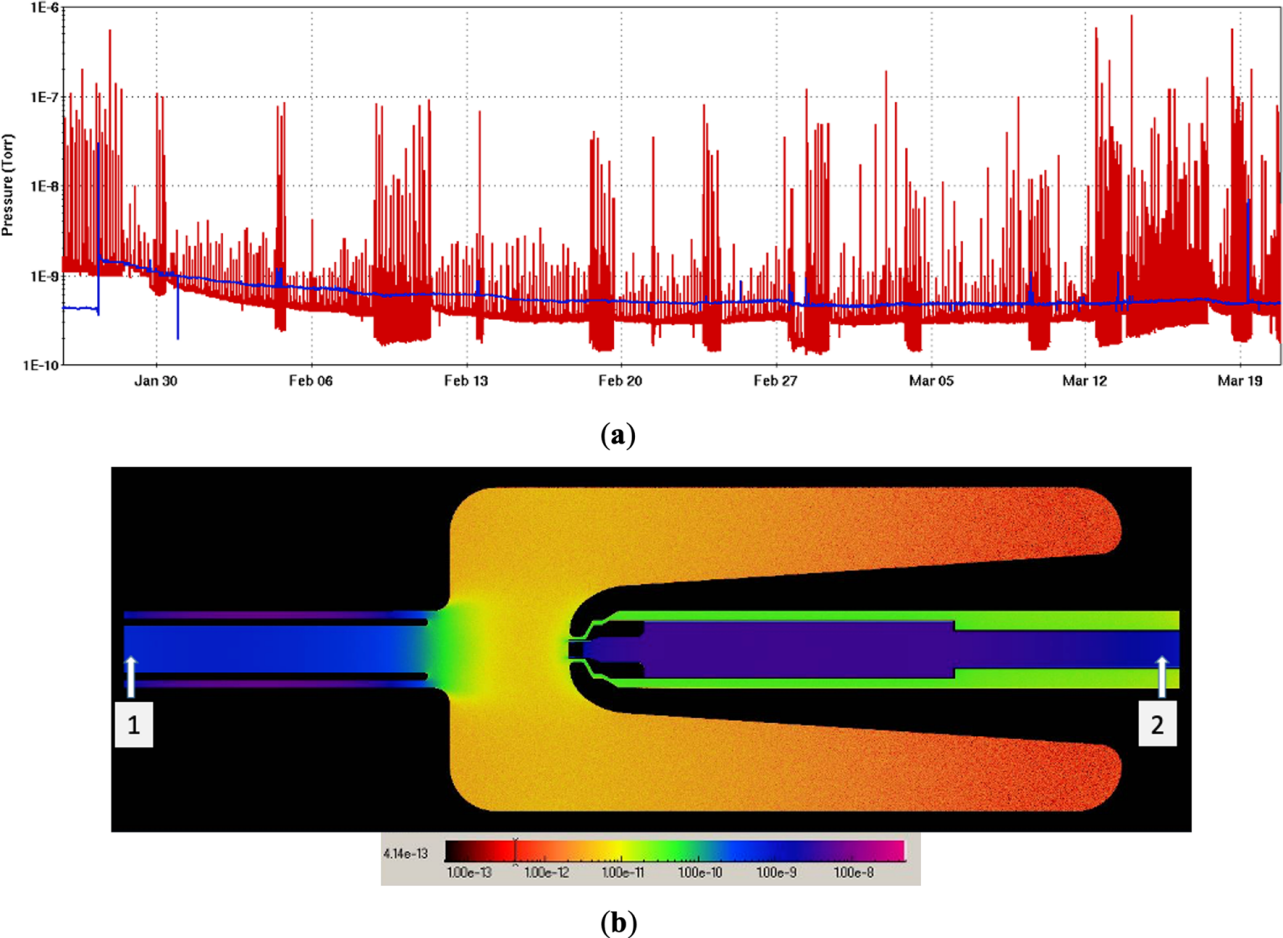
SRF gun pressure simulation. (**a**) The vacuum gauges reading from the FPC and cathode transfer chamber for long-term CW operation (blue: pressure in cathode stalk labeled 2; red: pressure in FPC labeled 1 in (**b**). (**b**) Gas molecule distributions in SRF gun simulated by Molflow+. The units in the color bar is torrs.

One of the significant advantages of the SRF gun is the entire 4 K cavity works as a very powerful vacuum cryo-pump. The 300 K cathode temperature is the hottest spot in the gun, avoiding any cold trapped gas on its surface. It is impossible to directly measure the total pressure and partial pressures of constituent gases inside the SRF gun; thus, we used Monte-Carlo simulation to map the pressure around the cathode. There are two cold cathode gauges close to the gun: one is at the fundamental power coupler (FPC) at the exit port of the gun, another one is installed in the cathode transfer system. Figure 6a shows the gauge reading from both locations. In the simulation, we used both gauges readings as boundary conditions and H_2_ sticking factor of 0.3 (e.g. within the typical range of 0.22–0.6) for the 4 K Nb wall. The majority of the gas load is originating from the FPC and beam pipe. Figure 6b shows the pressure distribution inside the gun simulated by Molflow+^32^. The simulation shows that base pressure at the cathode surface is about 2.3 × 10^–11^ torr with the H_2_O partial pressure under 10^–13^ torr: at these values the cathode should survive for years. It implies that the cathode lifetime in our SRF gun is dominated most likely by the transient multipacting during the ramp up of the RF power to the gun to the operational voltage.

Another concern about using semiconductor photocathode in SRF gun is cross-contamination between the cathode and gun cavity. Two major sources may contaminate the gun: one is the particles introduced during cathode insertion, the second one is the cathode flakes dropping into the SRF gun during MP or gun operation. Since the observed radiation level in RF CW operation did not increase before and after swapping the cathode, the cathode insertion did not contribute to the field emission. However, we did observe the radiation increase over a year of gun operation indicating gradual degradation in gun performance. This could be caused by migration of small amounts of cathode material into the gun. The gun performance is reestablished to the normal working voltage by helium processing on as-needed-basis^33^, typically one helium processing in a few years.

We extracted electron bunches with charge exceeding 13 nC from our SRF gun—in fact the measurements were limited by saturation of the integrated current transformer (ICT) used in the CeC accelerator. While this is the highest charge generated from the SRF gun with a photocathode, our simulations show that if necessary, our SRF gun and the high QE cathodes have the capability to operate well beyond this level.

**Figure 7:**
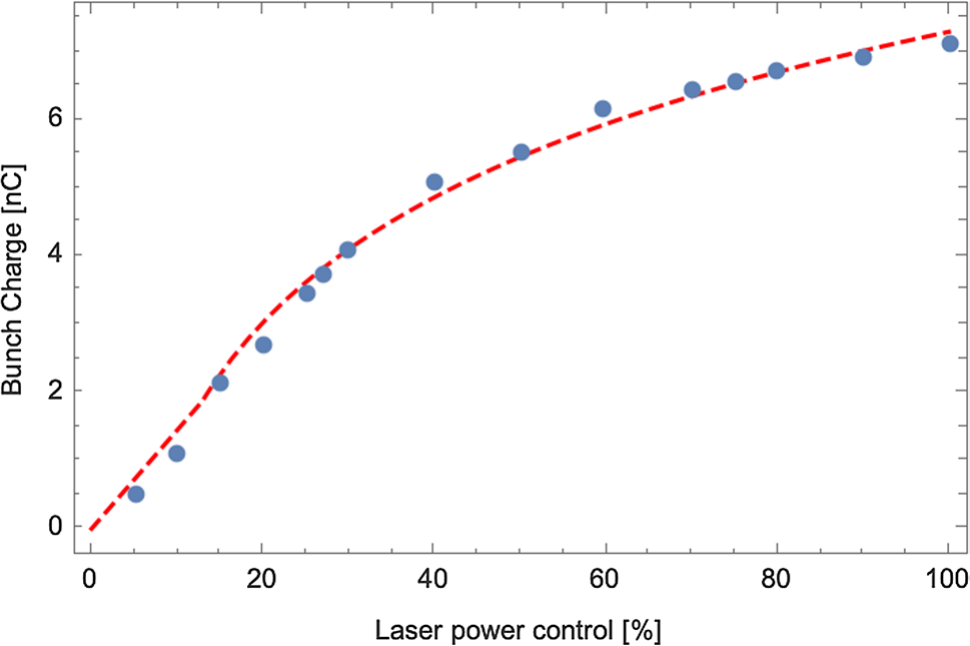
Space charge limit of extracting high bunch charges. The bunch charge extracted from the gun as the function of percentage of the laser power. The dash line is derived from the space charge limit model. The dots are from the experiments.

Two possible mechanisms may limit the extractable charge from the electron source: the surface charge limit and the space charge limit. The surface charge limit is usually observed in a bulk semiconductor photocathode such as GaAs and diamond cathode^34–36^. In this process, electrons trapped at the surface create a negative potential that inhibits the photo-excited electrons in the conduction band from diffusing to the surface and escaping the cathode. This creates temporal distortion in the emitted charge, with more charge emitted at the start of the pulse and much less thereafter, limiting the total bunch charge from the surface. We measured the bunch charge as the function of laser power shown in Fig. 7. The gun voltage is 1.25 MV which corresponds to the 12.5 MV/m gradient on the recessed cathode. The laser pulse length is 375 ps, and the laser spot is 6 mm in diameter. Using above parameters and gaussian laser shape with half-sigma of the laser diameter, we obtained the dashed curve that matches well to the measured data follow the reference^37^. Thus, the bunch space charge limit can be fully explained. We did not observe the surface charge limit of K–Cs–Sb photocathode up to 13 nC. By increasing the laser spot size and the bunch length up to 1 ns, we should be able to extract the bunch charge from exceeding 13 nC.

## Conclusion

The combination of the QWR SRF gun with high quantum efficiency, low thermal emittance K–Cs–Sb photocathode has demonstrated delivery of high charge, low emittance electron beam with long operational lifetime. Photocathodes fabricated using our unique fast growth recipe show exceptional performance, including long lifetime and generation of CW electron beams with record low emittances. With the large cryo-pumping speed of the SRF cavity, the cathode can typically sustain a month (or sometime even longer) of continuous operation. Many challenging issues related to integrating room temperature, high QE photocathode into the SRF gun have been investigated and successfully resolved.

## Data Availability

The datasets generated during and/or analysed during the current study are available from the corresponding author on reasonable request.
